# Octa­kis(dimethyl sulfoxide-κ*O*)cerium(III) μ_6_-oxido-dodeca-μ_2_-oxido-hexa­oxidohexa­molybdate(VI) dimethyl sulfoxide tetrasolvate

**DOI:** 10.1107/S1600536812025949

**Published:** 2012-06-16

**Authors:** Arbia Ben Khélifa, Michel Giorgi, Mohamed Salah Belkhiria

**Affiliations:** a Laboratoire de Physico-Chimie des Matériaux, Université de Monastir, Faculté des Sciences de Monastir, Tunisia; b Spectropole, Université d ’Aix-Marseille, Faculté des Science St-Jérôme, Avenue Escadrille Normandie-Niemen, 13397 Marseille cedex 20, France

## Abstract

The title complex, [Ce(C_2_H_6_OS)_8_]_2_[Mo_6_O_19_]_3_·4C_2_H_6_OS, was obtained as a byproduct of the reaction of [(C_4_H_9_)_4_N]_2_[Mo_6_O_19_] with Ce(NO_3_)_3_·6H_2_O and phthalic acid in dimethyl­sulfoxide solution. The asymmetric unit consists of a complex [Ce(C_2_H_6_OS)_8_]^3+^ cation, one and a half of the Lindqvist-type [Mo_6_O_19_]^2−^ polyanions and two dimethyl­sulfoxide solvent mol­ecules; the half polyanion lies on an inversion center. The Ce^3+^ ion is coordinated by eight dimethyl­sulfoxide ligands through the O atoms in the form of a distorted square antiprism. The Ce—O bond lengths range from 2.429 (6) to 2.550 (5) Å. The cohesion of the structure is ensured by S⋯O [3.115 (6), 3.242 (10) and 3.12 (3) Å], O⋯O [3.037 (10) Å] and C—H⋯O inter­actions between cations and anions. The S and C atoms of a dmso ligand are disordered over three sites in a 0.45:0.30:0.25 ratio. The dimethyl­sulfoxide solvent mol­ecules are highly disordered and could not be modelled successfully; their contribution was therefore removed from the refinement using the SQUEEZE routine in *PLATON* [Spek (2009 ▶). *Acta Cryst.* D**65**, 148–155]. Potential solvent-accessible voids of 500.0 Å^3^ occur in the crystal structure.

## Related literature

For general background, physical properties and applications of polyoxidometalates, see: Dolbecq *et al.* (2010 ▶). For the synthesis of [(C_4_H_9_)_4_N]_2_[Mo_6_O_19_], see: Hur *et al.* (1990 ▶). For related structures, see: Wang *et al.* (2003 ▶); Koo & Lee (2006 ▶); Qiu *et al.* (2006 ▶). For crystallographic analysis, see: Spek (2009 ▶).
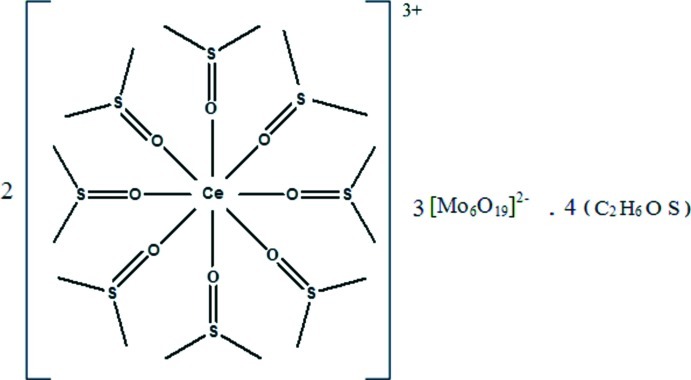


## Experimental

### 

#### Crystal data

[Ce(C_2_H_6_OS)_8_]_2_[Mo_6_O_19_]_3_·4C_2_H_6_OS
*M* *_r_* = 4481.72
Triclinic, 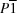

*a* = 13.4590 (2) Å
*b* = 15.4688 (3) Å
*c* = 17.6599 (4) Å
α = 90.281 (1)°
β = 98.468 (1)°
γ = 115.580 (1)°
*V* = 3270.48 (11) Å^3^
*Z* = 1
Mo *K*α radiation
μ = 2.75 mm^−1^
*T* = 223 K
0.20 × 0.16 × 0.08 mm

#### Data collection

Bruker–Nonius KappaCCD diffractometer
Absorption correction: multi-scan (*SORTAV*; Blessing, 1995 ▶) *T*_min_ = 0.691, *T*_max_ = 0.739
69065 measured reflections
12827 independent reflections
10059 reflections with *I* > 2σ(*I*)
*R*_int_ = 0.063

#### Refinement

*R*[*F* ^2^ > 2σ(*F* ^2^)] = 0.060
*wR*(*F* ^2^) = 0.159
*S* = 1.04
12827 reflections
680 parameters
H-atom parameters constrained
Δρ_max_ = 2.47 e Å^−3^
Δρ_min_ = −1.81 e Å^−3^

### 

Data collection: *COLLECT* (Nonius, 2002 ▶); cell refinement: *HKL*-*DENZO*/*SCALEPACK* (Otwinowski & Minor, 1997 ▶); data reduction: *HKL*-*DENZO*/*SCALEPACK*; program(s) used to solve structure: *SIR2004* (Burla *et al.*, 2005 ▶); program(s) used to refine structure: *SHELXL97* (Sheldrick, 2008 ▶); molecular graphics: *ORTEP-3* (Farrugia, 1997 ▶); software used to prepare material for publication: *SHELXL97*.

## Supplementary Material

Crystal structure: contains datablock(s) I, global. DOI: 10.1107/S1600536812025949/pv2546sup1.cif

Structure factors: contains datablock(s) I. DOI: 10.1107/S1600536812025949/pv2546Isup2.hkl

Additional supplementary materials:  crystallographic information; 3D view; checkCIF report

## Figures and Tables

**Table 1:** Hydrogen-bond geometry (Å, °)

*D*—H⋯*A*	*D*—H	H⋯*A*	*D*⋯*A*	*D*—H⋯*A*
C2—H2*B*⋯O13^i^	0.96	2.32	3.030 (14)	130
C6—H6*B*⋯O2^ii^	0.96	2.41	3.291 (18)	153
C11—H11*A*⋯O10	0.96	2.50	3.437 (15)	166
C14—H14*A*⋯O5^iii^	0.96	2.46	3.386 (19)	163
C14—H14*B*⋯O20^iv^	0.96	2.36	3.255 (17)	154
C16—H16*C*⋯O14^iii^	0.96	2.53	3.420 (17)	154

Symmetry codes: (i) 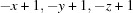; (ii) 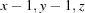; (iii) 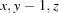; (iv) 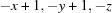.

**Fig. 1.**
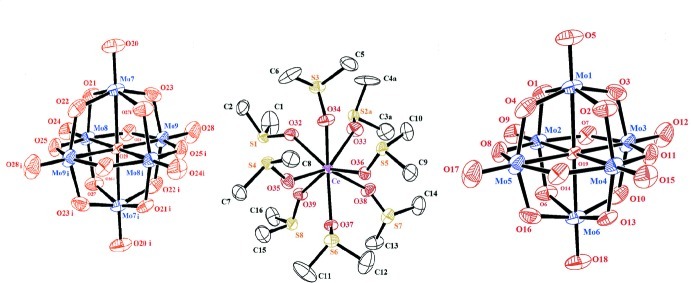
Molecular structure of the cation and anion complexes of the title compound. Displacement ellipsoids are drawn at the 30% probability level for non hydrogen atoms.

**Fig. 2.**
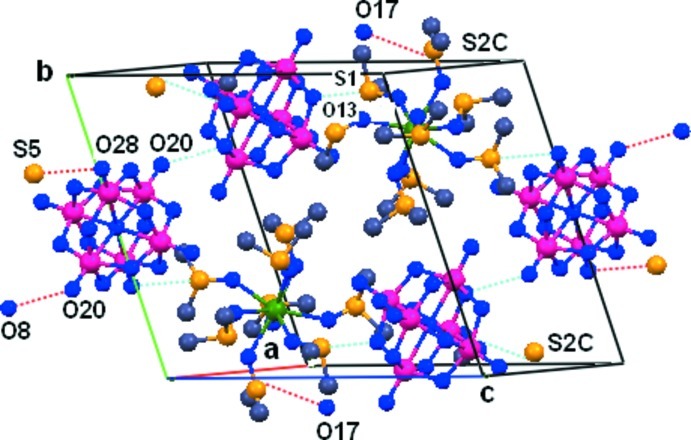
The lattice framework of the title compound, showing supramolecular ring-like clusters assembly via non-typical S···O and O···O contact interactions. The C—H···O hydrogen bonds are omitted for clarity.

## supplementary crystallographic information

### Comment

Polyoxidometalates (POM's) is an important class of molecular metal oxides in which early transition metal cations, bridged by oxide anions, form oligomeric aggregates. They have various chemical compositions and fascinating molecular structures (Dolbecq *et al.*, 2010). The title complex was obtained as a byproduct of the reaction of [(C_4_H_9_)_4_N]_2_[Mo_6_O_19_] with Ce(NO_3_)_3_.6H_2_O and phthalic acid in dimethylsulfoxide solution.

The asymmetric unit of the title compound contains one and a half of the Lindqvist-type [Mo_6_O_19_]^2-^ polyanion, one [Ce(dmso)_8_]^3+^ cation and two dimethylsulfoxide solvent molecules. The S2 and C3/C4 atoms of a dmso ligand were disordered over three sites in 0.45:0.30:0.25 ratio. There are two dimethylsulfoxide solvent molecules in an asymmetric unit which were disordered and were therefore, removed. The Ce^3+^ cation is octacoordinated to dimethylsulfoxide ligands through the oxygen atoms. The molecular structure of the cation and the anions of the title compound is presented in Figure 1. The Ce—O bond lengths, ranging from 2.429 (6) to 2.550 (5) Å, are typical for similar cerium complexes in Lindqvist-type polyoxidometalates (Wang *et al.*, 2003). The two crystallographically independant polyanions [Mo_6_O_19_]^2-^ are both constructed of six [MoO_6_] distorted octahedra sharing common edges and one common vertex at the central O atoms. The latters are respectively located on general and special positions. The Mo —O bond lengths, ranging from 1.671 (7) to 2.321 (5) Å, agree with those reported for [Mo_6_O_19_]^2-^ polyanions (Koo & Lee, 2006).

The cations and anions of the structure are interlinked through contact interactions and form supramolecular cluster anions assembly as shown in Figure 2. The cluster of anions are connected with the cation, through strong non typical contact interactions between sulfur atoms S1, S5 and S2C of the dmso ligand and oxygen atoms O13, O17 and O28 of cluster anions with interatomic distances O13···S1^i^, 3.115 (6) Å, O28···S5, 3.242 (10) Å and O17···S2C^ii^, 3.12 (3) Å. In addition, the cluster anions are directly interlinked through bridged and terminal oxygen atoms respectively O8 and O20 with interatomic distance O8···O20, 3.037 (10) Å (Koo & Lee, 2006; Qiu *et al.*, 2006); symmetry codes: (i) -*x* + 1, -*y* + 1, -*z* + 1; (ii) *x*, *y* + 1, *z*.

### Experimental

The [(C_4_H_9_)_4_N]_2_[Mo_6_O_19_] salt was synthesized as described in the literature (Hur *et al.*, 1990). A dimethylsulfoxide solution of Ce(NO_3_)_3_.6H_2_O (1 mmol, 0.433 g dissolved in 3 ml) was added dropwise to a yellow dmso solution of [(Bu)_4_N]_2_[Mo_6_O_19_] (0.2 mmol, 0.273 g dissolved in 13 ml). The resulting mixture was heated under stirring at 333 K for about 1 h. Then, phthalic acid (1 mmol, 0.166 g dissolved in 4 ml dmso) was added to the reaction mixture, followed by stirring and heating at 333 K for 1 h. Single crystals of the title compound, suitable for X-ray crystallographic studies, were obtained by diffusion of 2-propanol through the dimethylsulfoxide solution.

### Refinement

The two dimethylsulfoxide solvent molecules of the asymmetric unit were disordered and were therefore removed by the command SQUEEZE of *PLATON* (Spek, 2009). The solvent-free model was employed for the final refinement. All H atoms were refined using a riding model with C—H = 0.96 Å and *U_iso_*(H) = 1.5 *U_eq_*(C) and were allowed to rotate freely around the C—C bond except those bound to disordered carbon atoms. The S2 and C3/C4 atoms of a dmso ligand were disordered over three sites in 0.45:0.30:0.25 ratio which were modeled with the commands EADP and EXYZ in *SHELXL-97*.

### Crystal data

**Table:**

[Ce(C_2_H_6_OS)_8_]_2_[Mo_6_O_19_]_3_·4C_2_H_6_OS	*Z* = 1
*M**_r_* = 4481.72	*F*(000) = 2168
Triclinic, *P*1	*D*_x_ = 2.117 Mg m^−^^3^
Hall symbol: -P 1	Mo *K*α radiation, λ = 0.71073 Å
*a* = 13.4590 (2) Å	Cell parameters from 80805 reflections
*b* = 15.4688 (3) Å	θ = 2.3–29.9°
*c* = 17.6599 (4) Å	µ = 2.75 mm^−^^1^
α = 90.281 (1)°	*T* = 223 K
β = 98.468 (1)°	Prism, yellow
γ = 115.580 (1)°	0.20 × 0.16 × 0.08 mm
*V* = 3270.48 (11) Å^3^	

### Data collection

**Table:**

Bruker–Nonius KappaCCD diffractometer	12827 independent reflections
Radiation source: fine-focus sealed tube	10059 reflections with *I* > 2σ(*I*)
Graphite monochromator	*R*_int_ = 0.063
φ & ω scans	θ_max_ = 27.5°, θ_min_ = 2.9°
Absorption correction: multi-scan (*SORTAV*; Blessing, 1995)	*h* = −16→16
*T*_min_ = 0.691, *T*_max_ = 0.739	*k* = −19→19
69065 measured reflections	*l* = −21→21

### Refinement

**Table:**

Refinement on *F*^2^	Primary atom site location: structure-invariant direct methods
Least-squares matrix: full	Secondary atom site location: difference Fourier map
*R*[*F*^2^ > 2σ(*F*^2^)] = 0.060	Hydrogen site location: inferred from neighbouring sites
*wR*(*F*^2^) = 0.159	H-atom parameters constrained
*S* = 1.04	*w* = 1/[σ^2^(*F*_o_^2^) + (0.082*P*)^2^ + 11.5151*P*] where *P* = (*F*_o_^2^ + 2*F*_c_^2^)/3
12827 reflections	(Δ/σ)_max_ < 0.001
680 parameters	Δρ_max_ = 2.47 e Å^−^^3^
0 restraints	Δρ_min_ = −1.81 e Å^−^^3^

### Special details

**Table:**

Geometry. All s.u.'s (except the s.u. in the dihedral angle between two l.s. planes) are estimated using the full covariance matrix. The cell s.u.'s are taken into account individually in the estimation of s.u.'s in distances, angles and torsion angles; correlations between s.u.'s in cell parameters are only used when they are defined by crystal symmetry. An approximate (isotropic) treatment of cell s.u.'s is used for estimating s.u.'s involving l.s. planes.
Refinement. Refinement of *F*^2^ against ALL reflections. The weighted *R*-factor *wR* and goodness of fit *S* are based on *F*^2^, conventional *R*-factors *R* are based on *F*, with *F* set to zero for negative *F*^2^. The threshold expression of *F*^2^ > 2σ(*F*^2^) is used only for calculating *R*-factors(gt) *etc*. and is not relevant to the choice of reflections for refinement. *R*-factors based on *F*^2^ are statistically about twice as large as those based on *F*, and *R*- factors based on ALL data will be even larger.

### Fractional atomic coordinates and isotropic or equivalent isotropic displacement parameters (Å^2^)

**Table:**

	*x*	*y*	*z*	*U*_iso_*/*U*_eq_	Occ. (<1)
Ce1	0.37882 (3)	0.20892 (3)	0.24041 (2)	0.03716 (12)	
Mo1	0.67824 (6)	0.92294 (5)	0.16090 (4)	0.04819 (18)	
Mo2	0.54384 (6)	0.69998 (5)	0.19987 (4)	0.05286 (19)	
Mo8	−0.00354 (7)	0.45025 (6)	0.12342 (4)	0.0570 (2)	
Mo4	0.77556 (7)	0.98658 (5)	0.34389 (4)	0.0617 (2)	
Mo7	0.18589 (6)	0.60787 (6)	0.03598 (5)	0.0629 (2)	
Mo6	0.64036 (7)	0.76231 (5)	0.38286 (4)	0.0564 (2)	
Mo3	0.81409 (6)	0.81761 (6)	0.25863 (5)	0.0596 (2)	
Mo5	0.50644 (7)	0.87007 (7)	0.28498 (5)	0.0656 (2)	
Mo9	−0.05413 (8)	0.61545 (6)	0.03477 (5)	0.0679 (2)	
S1	0.24857 (17)	0.08769 (17)	0.40375 (12)	0.0534 (5)	
S2A	0.2195 (8)	−0.0474 (7)	0.1700 (6)	0.062 (2)	0.45
C3A	0.3010 (13)	−0.0760 (10)	0.1039 (10)	0.107 (4)	0.45
H3A1	0.2621	−0.0869	0.0521	0.160*	0.45
H3A2	0.3088	−0.1327	0.1188	0.160*	0.45
H3A3	0.3736	−0.0230	0.1072	0.160*	0.45
C4A	0.0865 (19)	−0.097 (2)	0.107 (3)	0.142 (19)	0.45
H4A1	0.0373	−0.0755	0.1263	0.213*	0.45
H4A2	0.0545	−0.1662	0.1055	0.213*	0.45
H4A3	0.0967	−0.0769	0.0566	0.213*	0.45
S2B	0.2032 (7)	−0.0376 (6)	0.1133 (5)	0.0618 (19)	0.30
C3B	0.3010 (13)	−0.0760 (10)	0.1039 (10)	0.107 (4)	0.30
H3B1	0.3444	−0.0410	0.0663	0.160*	0.30
H3B2	0.2651	−0.1434	0.0878	0.160*	0.30
H3B3	0.3491	−0.0656	0.1523	0.160*	0.30
C4B	0.151 (3)	−0.121 (2)	0.186 (2)	0.133 (12)	0.30
H4B1	0.0904	−0.1135	0.2026	0.200*	0.30
H4B2	0.2101	−0.1072	0.2284	0.200*	0.30
H4B3	0.1256	−0.1853	0.1642	0.200*	0.30
S2C	0.2722 (17)	−0.0281 (16)	0.1755 (13)	0.083 (6)	0.25
C3C	0.3010 (13)	−0.0760 (10)	0.1039 (10)	0.107 (4)	0.25
H3C1	0.3707	−0.0312	0.0906	0.160*	0.25
H3C2	0.2428	−0.0913	0.0604	0.160*	0.25
H3C3	0.3065	−0.1336	0.1188	0.160*	0.25
C4C	0.151 (3)	−0.121 (2)	0.186 (2)	0.133 (12)	0.25
H4C1	0.1204	−0.1034	0.2256	0.200*	0.25
H4C2	0.1642	−0.1754	0.1989	0.200*	0.25
H4C3	0.0997	−0.1361	0.1383	0.200*	0.25
S3	0.07310 (18)	0.13552 (17)	0.20031 (15)	0.0617 (6)	
S4	0.30340 (18)	0.37390 (15)	0.34142 (14)	0.0560 (5)	
S5	0.30121 (18)	0.31060 (16)	0.06817 (12)	0.0511 (5)	
S6	0.60406 (19)	0.45084 (16)	0.24827 (15)	0.0590 (5)	
S7	0.5942 (2)	0.22013 (18)	0.13221 (16)	0.0656 (6)	
S8	0.5973 (2)	0.2100 (2)	0.39721 (14)	0.0680 (6)	
O1	0.5842 (5)	0.7923 (4)	0.1237 (3)	0.0553 (14)	
O2	0.7666 (6)	1.0238 (4)	0.2402 (4)	0.0665 (17)	
O3	0.8010 (5)	0.8888 (4)	0.1729 (3)	0.0564 (14)	
O4	0.5505 (6)	0.9284 (5)	0.1907 (4)	0.0659 (17)	
O5	0.6949 (6)	0.9812 (5)	0.0800 (4)	0.0744 (19)	
O6	0.5536 (5)	0.6627 (4)	0.3044 (4)	0.0633 (16)	
O7	0.6927 (6)	0.7071 (5)	0.2042 (4)	0.0648 (16)	
O8	0.4430 (5)	0.7471 (5)	0.2238 (4)	0.0658 (17)	
O9	0.4633 (7)	0.5952 (5)	0.1479 (4)	0.084 (2)	
O10	0.7699 (5)	0.7586 (5)	0.3529 (4)	0.0665 (17)	
O11	0.8772 (5)	0.9393 (5)	0.3200 (4)	0.0699 (18)	
O12	0.9270 (7)	0.8005 (8)	0.2518 (6)	0.105 (3)	
O13	0.7362 (6)	0.8943 (4)	0.4187 (3)	0.0646 (17)	
O14	0.6301 (7)	0.9819 (5)	0.3400 (4)	0.079 (2)	
O15	0.8613 (8)	1.0899 (5)	0.3958 (5)	0.102 (3)	
O16	0.5197 (6)	0.7980 (5)	0.3720 (4)	0.0687 (18)	
O17	0.3937 (8)	0.8861 (8)	0.2945 (6)	0.115 (3)	
O18	0.6276 (8)	0.7041 (6)	0.4632 (4)	0.093 (3)	
O19	0.6601 (4)	0.8432 (3)	0.2719 (3)	0.0357 (10)	
O20	0.3191 (6)	0.6918 (6)	0.0598 (5)	0.097 (3)	
O21	0.1477 (5)	0.5494 (4)	0.1283 (3)	0.0607 (15)	
O22	0.1947 (6)	0.4962 (7)	0.0006 (4)	0.082 (2)	
O23	0.1046 (6)	0.6812 (4)	0.0549 (4)	0.0746 (19)	
O24	−0.0059 (7)	0.4147 (6)	0.2128 (4)	0.086 (2)	
O25	0.0428 (6)	0.3671 (4)	0.0728 (4)	0.0675 (17)	
O26	−0.0469 (6)	0.5526 (6)	0.1282 (3)	0.0725 (19)	
O27	−0.1511 (5)	0.3721 (5)	0.0729 (4)	0.074 (2)	
O28	−0.0966 (9)	0.6975 (7)	0.0588 (6)	0.112 (3)	
O29	0.0000	0.5000	0.0000	0.0404 (15)	
O32	0.2459 (5)	0.1094 (6)	0.3214 (4)	0.083 (2)	
O33	0.2578 (7)	0.0589 (5)	0.1608 (5)	0.088 (2)	
O34	0.1906 (5)	0.2087 (5)	0.1954 (4)	0.0674 (18)	
O35	0.3861 (6)	0.3394 (5)	0.3216 (4)	0.0667 (17)	
O36	0.3887 (5)	0.3088 (4)	0.1325 (3)	0.0561 (14)	
O37	0.5755 (4)	0.3460 (4)	0.2596 (4)	0.0529 (13)	
O38	0.4921 (5)	0.1623 (5)	0.1676 (5)	0.0730 (19)	
O39	0.4834 (5)	0.1710 (5)	0.3498 (4)	0.0627 (16)	
C1	0.215 (2)	−0.0349 (12)	0.4032 (12)	0.157 (9)	
H1A	0.1498	−0.0702	0.3657	0.235*	
H1B	0.2007	−0.0566	0.4531	0.235*	
H1C	0.2764	−0.0451	0.3907	0.235*	
C2	0.1235 (10)	0.0805 (15)	0.4247 (7)	0.118 (6)	
H2A	0.1006	0.1209	0.3928	0.178*	
H2B	0.1333	0.1014	0.4777	0.178*	
H2C	0.0673	0.0152	0.4153	0.178*	
C5	0.0023 (11)	0.1194 (16)	0.1039 (8)	0.147 (8)	
H5A	0.0192	0.1811	0.0841	0.221*	
H5B	−0.0768	0.0851	0.1028	0.221*	
H5C	0.0263	0.0834	0.0727	0.221*	
C6	0.0146 (11)	0.2028 (11)	0.2400 (12)	0.122 (6)	
H6A	0.0433	0.2170	0.2940	0.184*	
H6B	−0.0651	0.1667	0.2325	0.184*	
H6C	0.0336	0.2618	0.2154	0.184*	
C7	0.3831 (9)	0.4713 (7)	0.4113 (6)	0.071 (3)	
H7A	0.4101	0.4482	0.4563	0.107*	
H7B	0.3371	0.5004	0.4245	0.107*	
H7C	0.4453	0.5180	0.3909	0.107*	
C8	0.2828 (10)	0.4397 (8)	0.2643 (7)	0.083 (3)	
H8A	0.3534	0.4903	0.2575	0.124*	
H8B	0.2340	0.4669	0.2752	0.124*	
H8C	0.2499	0.3977	0.2182	0.124*	
C9	0.3816 (10)	0.3976 (8)	0.0101 (6)	0.079 (3)	
H9A	0.4085	0.4606	0.0354	0.119*	
H9B	0.3361	0.3936	−0.0383	0.119*	
H9C	0.4438	0.3861	0.0017	0.119*	
C10	0.2637 (11)	0.2072 (9)	0.0099 (6)	0.079 (3)	
H10A	0.3298	0.2024	0.0007	0.118*	
H10B	0.2216	0.2098	−0.0382	0.118*	
H10C	0.2191	0.1520	0.0348	0.118*	
C11	0.7005 (16)	0.5176 (9)	0.3288 (10)	0.134 (7)	
H11A	0.7070	0.5819	0.3307	0.201*	
H11B	0.7720	0.5195	0.3255	0.201*	
H11C	0.6755	0.4881	0.3745	0.201*	
C12	0.6920 (16)	0.4827 (9)	0.1786 (10)	0.120 (6)	
H12A	0.7466	0.4582	0.1902	0.180*	
H12B	0.7292	0.5514	0.1787	0.180*	
H12C	0.6484	0.4558	0.1289	0.180*	
C13	0.7069 (10)	0.2150 (10)	0.1954 (9)	0.094 (4)	
H13A	0.6795	0.1574	0.2220	0.141*	
H13B	0.7401	0.2700	0.2320	0.141*	
H13C	0.7618	0.2149	0.1664	0.141*	
C14	0.5824 (14)	0.1392 (10)	0.0555 (8)	0.103 (4)	
H14A	0.6158	0.0981	0.0742	0.155*	
H14B	0.6201	0.1751	0.0158	0.155*	
H14C	0.5050	0.1008	0.0351	0.155*	
C15	0.6047 (13)	0.3028 (10)	0.4582 (8)	0.098 (4)	
H15A	0.5785	0.3432	0.4289	0.147*	
H15B	0.6806	0.3402	0.4824	0.147*	
H15C	0.5589	0.2759	0.4967	0.147*	
C16	0.5840 (13)	0.1237 (10)	0.4661 (7)	0.096 (4)	
H16A	0.5092	0.0958	0.4772	0.143*	
H16B	0.6357	0.1546	0.5124	0.143*	
H16C	0.6000	0.0742	0.4460	0.143*	

### Atomic displacement parameters (Å^2^)

**Table:**

	*U*^11^	*U*^22^	*U*^33^	*U*^12^	*U*^13^	*U*^23^
Ce1	0.0368 (2)	0.0356 (2)	0.0368 (2)	0.01406 (17)	0.00461 (15)	0.00387 (15)
Mo1	0.0585 (4)	0.0430 (4)	0.0388 (4)	0.0190 (3)	0.0054 (3)	0.0099 (3)
Mo2	0.0586 (4)	0.0373 (4)	0.0421 (4)	0.0049 (3)	−0.0013 (3)	−0.0038 (3)
Mo8	0.0630 (4)	0.0590 (4)	0.0385 (4)	0.0203 (4)	−0.0025 (3)	0.0123 (3)
Mo4	0.0741 (5)	0.0387 (4)	0.0449 (4)	0.0031 (3)	−0.0025 (3)	−0.0052 (3)
Mo7	0.0478 (4)	0.0588 (5)	0.0571 (5)	0.0057 (4)	−0.0093 (3)	0.0092 (4)
Mo6	0.0700 (5)	0.0473 (4)	0.0356 (4)	0.0119 (4)	0.0042 (3)	0.0096 (3)
Mo3	0.0480 (4)	0.0693 (5)	0.0680 (5)	0.0320 (4)	0.0080 (3)	0.0134 (4)
Mo5	0.0579 (5)	0.0801 (6)	0.0713 (5)	0.0390 (4)	0.0193 (4)	0.0070 (4)
Mo9	0.0907 (6)	0.0627 (5)	0.0598 (5)	0.0488 (5)	−0.0080 (4)	−0.0070 (4)
S1	0.0471 (10)	0.0615 (13)	0.0419 (10)	0.0161 (10)	0.0029 (8)	0.0059 (9)
S2A	0.074 (5)	0.044 (5)	0.054 (4)	0.011 (4)	0.015 (5)	−0.002 (3)
C3A	0.117 (11)	0.066 (7)	0.131 (12)	0.034 (7)	0.020 (9)	−0.008 (7)
C4A	0.039 (12)	0.076 (19)	0.27 (5)	0.005 (12)	−0.03 (2)	−0.08 (3)
S2B	0.077 (5)	0.059 (4)	0.040 (4)	0.024 (4)	−0.001 (4)	−0.008 (3)
C3B	0.117 (11)	0.066 (7)	0.131 (12)	0.034 (7)	0.020 (9)	−0.008 (7)
C4B	0.16 (3)	0.073 (17)	0.15 (3)	0.019 (19)	0.08 (3)	0.014 (17)
S2C	0.102 (13)	0.057 (10)	0.073 (9)	0.017 (11)	0.014 (12)	0.014 (7)
C3C	0.117 (11)	0.066 (7)	0.131 (12)	0.034 (7)	0.020 (9)	−0.008 (7)
C4C	0.16 (3)	0.073 (17)	0.15 (3)	0.019 (19)	0.08 (3)	0.014 (17)
S3	0.0461 (11)	0.0552 (12)	0.0770 (15)	0.0163 (10)	0.0080 (10)	0.0262 (11)
S4	0.0509 (11)	0.0455 (11)	0.0668 (14)	0.0143 (9)	0.0176 (10)	−0.0035 (9)
S5	0.0577 (11)	0.0559 (12)	0.0407 (10)	0.0245 (10)	0.0123 (9)	0.0128 (9)
S6	0.0571 (12)	0.0432 (11)	0.0741 (15)	0.0198 (10)	0.0099 (11)	0.0076 (10)
S7	0.0771 (15)	0.0561 (13)	0.0763 (16)	0.0358 (12)	0.0292 (13)	0.0122 (11)
S8	0.0561 (13)	0.1000 (19)	0.0481 (12)	0.0357 (13)	0.0042 (10)	0.0070 (12)
O1	0.062 (3)	0.055 (3)	0.032 (3)	0.013 (3)	−0.003 (2)	−0.001 (2)
O2	0.082 (4)	0.042 (3)	0.060 (4)	0.015 (3)	0.003 (3)	0.005 (3)
O3	0.052 (3)	0.063 (4)	0.050 (3)	0.019 (3)	0.017 (3)	0.008 (3)
O4	0.071 (4)	0.070 (4)	0.066 (4)	0.045 (4)	−0.006 (3)	0.004 (3)
O5	0.097 (5)	0.068 (4)	0.050 (4)	0.029 (4)	0.012 (3)	0.025 (3)
O6	0.075 (4)	0.042 (3)	0.050 (3)	0.006 (3)	0.004 (3)	0.006 (3)
O7	0.082 (4)	0.062 (4)	0.062 (4)	0.043 (4)	0.011 (3)	−0.003 (3)
O8	0.040 (3)	0.077 (4)	0.062 (4)	0.010 (3)	0.003 (3)	0.002 (3)
O9	0.105 (6)	0.043 (3)	0.061 (4)	−0.003 (4)	0.002 (4)	−0.011 (3)
O10	0.064 (4)	0.063 (4)	0.066 (4)	0.026 (3)	−0.007 (3)	0.014 (3)
O11	0.043 (3)	0.066 (4)	0.069 (4)	0.001 (3)	−0.010 (3)	0.008 (3)
O12	0.080 (5)	0.148 (9)	0.122 (7)	0.080 (6)	0.023 (5)	0.031 (6)
O13	0.080 (4)	0.054 (3)	0.032 (3)	0.007 (3)	−0.003 (3)	−0.002 (2)
O14	0.112 (6)	0.063 (4)	0.075 (5)	0.045 (4)	0.036 (4)	−0.003 (3)
O15	0.122 (7)	0.051 (4)	0.075 (5)	−0.010 (4)	−0.005 (5)	−0.023 (4)
O16	0.069 (4)	0.079 (4)	0.046 (3)	0.016 (3)	0.024 (3)	0.004 (3)
O17	0.089 (6)	0.157 (9)	0.139 (9)	0.082 (7)	0.046 (6)	0.020 (7)
O18	0.125 (7)	0.075 (5)	0.046 (4)	0.016 (5)	0.005 (4)	0.021 (3)
O19	0.036 (2)	0.029 (2)	0.032 (2)	0.0066 (19)	−0.0001 (19)	−0.0017 (18)
O20	0.060 (4)	0.094 (6)	0.088 (6)	−0.005 (4)	−0.014 (4)	0.017 (5)
O21	0.060 (3)	0.058 (4)	0.045 (3)	0.017 (3)	−0.017 (3)	0.004 (3)
O22	0.054 (4)	0.129 (7)	0.066 (4)	0.044 (4)	0.003 (3)	0.019 (4)
O23	0.098 (5)	0.034 (3)	0.070 (4)	0.016 (3)	−0.010 (4)	−0.002 (3)
O24	0.099 (5)	0.103 (6)	0.047 (4)	0.038 (5)	0.005 (4)	0.029 (4)
O25	0.095 (5)	0.050 (3)	0.055 (4)	0.037 (3)	−0.009 (3)	0.010 (3)
O26	0.096 (5)	0.098 (5)	0.037 (3)	0.056 (4)	0.006 (3)	−0.001 (3)
O27	0.057 (4)	0.070 (4)	0.067 (4)	0.004 (3)	0.003 (3)	0.027 (3)
O28	0.158 (9)	0.113 (7)	0.098 (6)	0.103 (7)	−0.013 (6)	−0.018 (5)
O29	0.043 (4)	0.036 (4)	0.034 (4)	0.013 (3)	−0.006 (3)	−0.002 (3)
O32	0.055 (4)	0.103 (6)	0.054 (4)	0.002 (4)	0.006 (3)	0.035 (4)
O33	0.085 (5)	0.037 (3)	0.118 (7)	0.003 (3)	0.016 (4)	−0.032 (4)
O34	0.040 (3)	0.068 (4)	0.095 (5)	0.023 (3)	0.013 (3)	0.036 (4)
O35	0.075 (4)	0.066 (4)	0.065 (4)	0.037 (3)	0.008 (3)	−0.008 (3)
O36	0.057 (3)	0.059 (3)	0.042 (3)	0.016 (3)	0.005 (2)	0.016 (3)
O37	0.044 (3)	0.037 (3)	0.066 (4)	0.007 (2)	0.009 (3)	0.007 (2)
O38	0.060 (4)	0.062 (4)	0.101 (5)	0.025 (3)	0.032 (4)	−0.005 (4)
O39	0.053 (3)	0.069 (4)	0.063 (4)	0.028 (3)	−0.005 (3)	0.015 (3)
C1	0.28 (3)	0.083 (10)	0.135 (15)	0.095 (15)	0.066 (16)	0.039 (10)
C2	0.074 (7)	0.24 (2)	0.052 (6)	0.083 (10)	0.008 (5)	0.006 (9)
C5	0.073 (8)	0.22 (2)	0.068 (8)	−0.002 (10)	−0.014 (6)	0.035 (10)
C6	0.064 (7)	0.109 (11)	0.203 (18)	0.030 (7)	0.071 (10)	0.033 (11)
C7	0.080 (7)	0.055 (5)	0.072 (6)	0.023 (5)	0.009 (5)	−0.012 (5)
C8	0.083 (7)	0.068 (7)	0.092 (8)	0.030 (6)	0.007 (6)	0.003 (6)
C9	0.091 (7)	0.069 (6)	0.062 (6)	0.019 (6)	0.019 (5)	0.031 (5)
C10	0.104 (8)	0.085 (7)	0.051 (5)	0.046 (7)	0.009 (5)	0.006 (5)
C11	0.170 (15)	0.057 (7)	0.117 (12)	0.021 (9)	−0.057 (11)	−0.010 (7)
C12	0.184 (16)	0.063 (7)	0.127 (12)	0.045 (9)	0.095 (12)	0.034 (8)
C13	0.064 (6)	0.090 (9)	0.128 (11)	0.034 (6)	0.014 (7)	0.032 (8)
C14	0.139 (12)	0.091 (9)	0.090 (9)	0.053 (9)	0.046 (9)	0.001 (7)
C15	0.116 (10)	0.086 (8)	0.076 (8)	0.033 (8)	0.006 (7)	0.004 (6)
C16	0.141 (11)	0.103 (9)	0.065 (7)	0.082 (9)	−0.009 (7)	0.008 (6)

### Geometric parameters (Å, º)

**Table:**

Ce1—O35	2.429 (6)	C4A—H4A3	0.9600
Ce1—O36	2.441 (5)	S2B—O33	1.526 (10)
Ce1—O39	2.445 (6)	S2B—C4B	1.81 (3)
Ce1—O32	2.453 (6)	C4B—H4B1	0.9600
Ce1—O38	2.453 (6)	C4B—H4B2	0.9600
Ce1—O33	2.459 (6)	C4B—H4B3	0.9600
Ce1—O34	2.539 (6)	S2C—O33	1.46 (3)
Ce1—O37	2.550 (5)	S3—O34	1.512 (6)
Mo1—O5	1.684 (6)	S3—C6	1.749 (15)
Mo1—O4	1.906 (7)	S3—C5	1.785 (13)
Mo1—O1	1.911 (6)	S4—O35	1.507 (7)
Mo1—O2	1.923 (6)	S4—C8	1.765 (12)
Mo1—O3	1.925 (6)	S4—C7	1.771 (10)
Mo1—O19	2.309 (4)	S5—O36	1.521 (6)
Mo2—O9	1.680 (6)	S5—C10	1.735 (12)
Mo2—O8	1.890 (7)	S5—C9	1.765 (9)
Mo2—O1	1.926 (6)	S6—O37	1.518 (6)
Mo2—O6	1.940 (6)	S6—C11	1.749 (13)
Mo2—O7	1.949 (7)	S6—C12	1.757 (13)
Mo2—O19	2.317 (4)	S7—O38	1.508 (7)
Mo8—O24	1.675 (6)	S7—C13	1.776 (12)
Mo8—O27	1.896 (6)	S7—C14	1.782 (13)
Mo8—O26	1.915 (7)	S8—O39	1.495 (6)
Mo8—O25	1.922 (7)	S8—C15	1.749 (14)
Mo8—O21	1.938 (6)	S8—C16	1.780 (12)
Mo8—O29	2.3153 (7)	O22—Mo9^i^	1.942 (8)
Mo4—O15	1.676 (7)	O25—Mo9^i^	1.938 (7)
Mo4—O11	1.901 (8)	O27—Mo7^i^	1.969 (7)
Mo4—O13	1.907 (6)	O29—Mo9^i^	2.3113 (8)
Mo4—O14	1.918 (8)	O29—Mo8^i^	2.3153 (7)
Mo4—O2	1.925 (6)	O29—Mo7^i^	2.3160 (7)
Mo4—O19	2.316 (4)	C1—H1A	0.9600
Mo7—O20	1.685 (7)	C1—H1B	0.9600
Mo7—O22	1.892 (9)	C1—H1C	0.9600
Mo7—O21	1.897 (6)	C2—H2A	0.9600
Mo7—O23	1.943 (8)	C2—H2B	0.9600
Mo7—O27^i^	1.969 (7)	C2—H2C	0.9600
Mo7—O29	2.3160 (7)	C5—H5A	0.9600
Mo6—O18	1.674 (7)	C5—H5B	0.9600
Mo6—O6	1.899 (6)	C5—H5C	0.9600
Mo6—O16	1.915 (7)	C6—H6A	0.9600
Mo6—O10	1.919 (7)	C6—H6B	0.9600
Mo6—O13	1.925 (6)	C6—H6C	0.9600
Mo6—O19	2.316 (4)	C7—H7A	0.9600
Mo3—O12	1.672 (7)	C7—H7B	0.9600
Mo3—O7	1.904 (7)	C7—H7C	0.9600
Mo3—O3	1.908 (6)	C8—H8A	0.9600
Mo3—O11	1.941 (7)	C8—H8B	0.9600
Mo3—O10	1.948 (7)	C8—H8C	0.9600
Mo3—O19	2.314 (5)	C9—H9A	0.9600
Mo5—O17	1.671 (7)	C9—H9B	0.9600
Mo5—O14	1.931 (8)	C9—H9C	0.9600
Mo5—O16	1.935 (7)	C10—H10A	0.9600
Mo5—O4	1.944 (7)	C10—H10B	0.9600
Mo5—O8	1.954 (7)	C10—H10C	0.9600
Mo5—O19	2.321 (5)	C11—H11A	0.9600
Mo9—O28	1.676 (8)	C11—H11B	0.9600
Mo9—O23	1.903 (8)	C11—H11C	0.9600
Mo9—O26	1.930 (7)	C12—H12A	0.9600
Mo9—O25^i^	1.938 (7)	C12—H12B	0.9600
Mo9—O22^i^	1.942 (8)	C12—H12C	0.9600
Mo9—O29	2.3113 (8)	C13—H13A	0.9600
S1—O32	1.492 (7)	C13—H13B	0.9600
S1—C2	1.734 (11)	C13—H13C	0.9600
S1—C1	1.749 (15)	C14—H14A	0.9600
S2A—O33	1.514 (13)	C14—H14B	0.9600
S2A—C4A	1.80 (3)	C14—H14C	0.9600
S2A—C3A	1.884 (18)	C15—H15A	0.9600
C3A—H3A1	0.9600	C15—H15B	0.9600
C3A—H3A2	0.9600	C15—H15C	0.9600
C3A—H3A3	0.9600	C16—H16A	0.9600
C4A—H4A1	0.9600	C16—H16B	0.9600
C4A—H4A2	0.9600	C16—H16C	0.9600
			
O8···O20	3.037 (11)	O13···S1^ii^	3.115 (6)
O28···S5^i^	3.242 (12)	O17···S2C^iii^	3.12 (3)
			
O35—Ce1—O36	87.8 (2)	O33—S2B—C4B	101.6 (12)
O35—Ce1—O39	88.2 (2)	S2B—C4B—H4B1	109.5
O36—Ce1—O39	146.3 (2)	S2B—C4B—H4B2	109.5
O35—Ce1—O32	82.8 (3)	H4B1—C4B—H4B2	109.5
O36—Ce1—O32	140.4 (2)	S2B—C4B—H4B3	109.5
O39—Ce1—O32	71.9 (2)	H4B1—C4B—H4B3	109.5
O35—Ce1—O38	142.6 (2)	H4B2—C4B—H4B3	109.5
O36—Ce1—O38	80.2 (2)	O34—S3—C6	103.8 (5)
O39—Ce1—O38	82.9 (3)	O34—S3—C5	103.1 (6)
O32—Ce1—O38	127.6 (3)	C6—S3—C5	98.3 (10)
O35—Ce1—O33	146.0 (3)	O35—S4—C8	105.7 (5)
O36—Ce1—O33	93.3 (3)	O35—S4—C7	104.3 (5)
O39—Ce1—O33	108.2 (3)	C8—S4—C7	98.6 (6)
O32—Ce1—O33	74.9 (3)	O36—S5—C10	104.0 (5)
O38—Ce1—O33	70.6 (3)	O36—S5—C9	103.2 (5)
O35—Ce1—O34	77.2 (2)	C10—S5—C9	99.7 (6)
O36—Ce1—O34	69.5 (2)	O37—S6—C11	106.5 (5)
O39—Ce1—O34	141.4 (2)	O37—S6—C12	105.3 (5)
O32—Ce1—O34	71.0 (2)	C11—S6—C12	99.7 (10)
O38—Ce1—O34	129.1 (3)	O38—S7—C13	104.8 (5)
O33—Ce1—O34	71.5 (3)	O38—S7—C14	102.3 (6)
O35—Ce1—O37	70.7 (2)	C13—S7—C14	99.2 (8)
O36—Ce1—O37	72.73 (19)	O39—S8—C15	105.0 (6)
O39—Ce1—O37	74.4 (2)	O39—S8—C16	103.4 (6)
O32—Ce1—O37	137.3 (2)	C15—S8—C16	98.2 (6)
O38—Ce1—O37	71.9 (2)	Mo1—O1—Mo2	116.5 (3)
O33—Ce1—O37	141.7 (2)	Mo1—O2—Mo4	116.8 (3)
O34—Ce1—O37	130.61 (19)	Mo3—O3—Mo1	116.7 (3)
O5—Mo1—O4	104.1 (3)	Mo1—O4—Mo5	116.3 (3)
O5—Mo1—O1	103.2 (3)	Mo6—O6—Mo2	117.0 (3)
O4—Mo1—O1	87.7 (3)	Mo3—O7—Mo2	116.9 (3)
O5—Mo1—O2	103.1 (3)	Mo2—O8—Mo5	117.2 (3)
O4—Mo1—O2	86.9 (3)	Mo6—O10—Mo3	116.5 (3)
O1—Mo1—O2	153.7 (2)	Mo4—O11—Mo3	117.0 (3)
O5—Mo1—O3	101.8 (3)	Mo4—O13—Mo6	117.8 (3)
O4—Mo1—O3	154.1 (3)	Mo4—O14—Mo5	116.2 (3)
O1—Mo1—O3	87.0 (3)	Mo6—O16—Mo5	116.5 (3)
O2—Mo1—O3	86.6 (3)	Mo1—O19—Mo3	89.79 (16)
O5—Mo1—O19	178.5 (3)	Mo1—O19—Mo4	90.22 (15)
O4—Mo1—O19	77.4 (2)	Mo3—O19—Mo4	90.06 (15)
O1—Mo1—O19	77.11 (19)	Mo1—O19—Mo6	179.5 (2)
O2—Mo1—O19	76.6 (2)	Mo3—O19—Mo6	90.51 (16)
O3—Mo1—O19	76.7 (2)	Mo4—O19—Mo6	90.18 (15)
O9—Mo2—O8	104.9 (4)	Mo1—O19—Mo2	89.70 (15)
O9—Mo2—O1	103.7 (3)	Mo3—O19—Mo2	90.26 (16)
O8—Mo2—O1	88.4 (3)	Mo4—O19—Mo2	179.7 (2)
O9—Mo2—O6	103.4 (3)	Mo6—O19—Mo2	89.89 (15)
O8—Mo2—O6	87.5 (3)	Mo1—O19—Mo5	89.87 (16)
O1—Mo2—O6	152.7 (2)	Mo3—O19—Mo5	179.5 (2)
O9—Mo2—O7	102.1 (4)	Mo4—O19—Mo5	89.64 (16)
O8—Mo2—O7	153.0 (3)	Mo6—O19—Mo5	89.83 (16)
O1—Mo2—O7	85.8 (3)	Mo2—O19—Mo5	90.04 (15)
O6—Mo2—O7	85.7 (3)	Mo7—O21—Mo8	116.7 (3)
O9—Mo2—O19	178.0 (3)	Mo7—O22—Mo9^i^	116.7 (3)
O8—Mo2—O19	77.0 (2)	Mo9—O23—Mo7	117.0 (3)
O1—Mo2—O19	76.6 (2)	Mo8—O25—Mo9^i^	116.2 (3)
O6—Mo2—O19	76.2 (2)	Mo8—O26—Mo9	116.4 (3)
O7—Mo2—O19	76.0 (2)	Mo8—O27—Mo7^i^	116.4 (3)
O24—Mo8—O27	102.8 (3)	Mo9—O29—Mo9^i^	180.00 (4)
O24—Mo8—O26	102.9 (4)	Mo9—O29—Mo8^i^	90.16 (3)
O27—Mo8—O26	87.2 (3)	Mo9^i^—O29—Mo8^i^	89.84 (3)
O24—Mo8—O25	103.2 (4)	Mo9—O29—Mo8	89.84 (3)
O27—Mo8—O25	87.7 (3)	Mo9^i^—O29—Mo8	90.16 (3)
O26—Mo8—O25	153.9 (3)	Mo8^i^—O29—Mo8	180.00 (4)
O24—Mo8—O21	103.4 (3)	Mo9—O29—Mo7	90.27 (4)
O27—Mo8—O21	153.7 (3)	Mo9^i^—O29—Mo7	89.73 (4)
O26—Mo8—O21	86.5 (3)	Mo8^i^—O29—Mo7	90.36 (3)
O25—Mo8—O21	86.8 (3)	Mo8—O29—Mo7	89.64 (3)
O24—Mo8—O29	179.8 (4)	Mo9—O29—Mo7^i^	89.73 (4)
O27—Mo8—O29	77.30 (19)	Mo9^i^—O29—Mo7^i^	90.27 (4)
O26—Mo8—O29	76.98 (19)	Mo8^i^—O29—Mo7^i^	89.64 (3)
O25—Mo8—O29	76.94 (18)	Mo8—O29—Mo7^i^	90.36 (3)
O21—Mo8—O29	76.45 (17)	Mo7—O29—Mo7^i^	180.0
O15—Mo4—O11	102.0 (4)	S1—O32—Ce1	137.2 (4)
O15—Mo4—O13	103.8 (4)	S2C—O33—S2B	51.3 (9)
O11—Mo4—O13	87.8 (3)	S2C—O33—Ce1	119.9 (10)
O15—Mo4—O14	103.9 (4)	S2A—O33—Ce1	136.0 (7)
O11—Mo4—O14	154.1 (3)	S2B—O33—Ce1	168.8 (6)
O13—Mo4—O14	86.8 (3)	S3—O34—Ce1	131.5 (3)
O15—Mo4—O2	103.6 (4)	S4—O35—Ce1	136.2 (4)
O11—Mo4—O2	87.4 (3)	S5—O36—Ce1	133.1 (3)
O13—Mo4—O2	152.5 (3)	S6—O37—Ce1	125.4 (3)
O14—Mo4—O2	85.7 (3)	S7—O38—Ce1	132.4 (4)
O15—Mo4—O19	178.9 (4)	S8—O39—Ce1	140.7 (4)
O11—Mo4—O19	76.8 (2)	S1—C1—H1A	109.5
O13—Mo4—O19	76.2 (2)	S1—C1—H1B	109.5
O14—Mo4—O19	77.2 (2)	H1A—C1—H1B	109.5
O2—Mo4—O19	76.4 (2)	S1—C1—H1C	109.5
O20—Mo7—O22	105.6 (4)	H1A—C1—H1C	109.5
O20—Mo7—O21	105.0 (3)	H1B—C1—H1C	109.5
O22—Mo7—O21	88.7 (3)	S1—C2—H2A	109.5
O20—Mo7—O23	101.3 (4)	S1—C2—H2B	109.5
O22—Mo7—O23	153.0 (3)	H2A—C2—H2B	109.5
O21—Mo7—O23	87.1 (3)	S1—C2—H2C	109.5
O20—Mo7—O27^i^	101.8 (3)	H2A—C2—H2C	109.5
O22—Mo7—O27^i^	86.6 (3)	H2B—C2—H2C	109.5
O21—Mo7—O27^i^	153.1 (3)	S3—C5—H5A	109.5
O23—Mo7—O27^i^	85.2 (3)	S3—C5—H5B	109.5
O20—Mo7—O29	176.4 (3)	H5A—C5—H5B	109.5
O22—Mo7—O29	77.2 (2)	S3—C5—H5C	109.5
O21—Mo7—O29	77.18 (17)	H5A—C5—H5C	109.5
O23—Mo7—O29	75.9 (2)	H5B—C5—H5C	109.5
O27^i^—Mo7—O29	75.94 (18)	S3—C6—H6A	109.5
O18—Mo6—O6	103.3 (3)	S3—C6—H6B	109.5
O18—Mo6—O16	103.5 (4)	H6A—C6—H6B	109.5
O6—Mo6—O16	87.7 (3)	S3—C6—H6C	109.5
O18—Mo6—O10	102.7 (4)	H6A—C6—H6C	109.5
O6—Mo6—O10	87.2 (3)	H6B—C6—H6C	109.5
O16—Mo6—O10	153.8 (3)	S4—C7—H7A	109.5
O18—Mo6—O13	103.9 (3)	S4—C7—H7B	109.5
O6—Mo6—O13	152.8 (2)	H7A—C7—H7B	109.5
O16—Mo6—O13	86.5 (3)	S4—C7—H7C	109.5
O10—Mo6—O13	86.3 (3)	H7A—C7—H7C	109.5
O18—Mo6—O19	179.3 (4)	H7B—C7—H7C	109.5
O6—Mo6—O19	76.9 (2)	S4—C8—H8A	109.5
O16—Mo6—O19	77.1 (2)	S4—C8—H8B	109.5
O10—Mo6—O19	76.7 (2)	H8A—C8—H8B	109.5
O13—Mo6—O19	75.9 (2)	S4—C8—H8C	109.5
O12—Mo3—O7	104.5 (4)	H8A—C8—H8C	109.5
O12—Mo3—O3	104.3 (4)	H8B—C8—H8C	109.5
O7—Mo3—O3	88.2 (3)	S5—C9—H9A	109.5
O12—Mo3—O11	102.5 (4)	S5—C9—H9B	109.5
O7—Mo3—O11	153.0 (3)	H9A—C9—H9B	109.5
O3—Mo3—O11	86.2 (3)	S5—C9—H9C	109.5
O12—Mo3—O10	102.6 (4)	H9A—C9—H9C	109.5
O7—Mo3—O10	87.3 (3)	H9B—C9—H9C	109.5
O3—Mo3—O10	153.0 (3)	S5—C10—H10A	109.5
O11—Mo3—O10	85.8 (3)	S5—C10—H10B	109.5
O12—Mo3—O19	178.3 (4)	H10A—C10—H10B	109.5
O7—Mo3—O19	76.9 (2)	S5—C10—H10C	109.5
O3—Mo3—O19	76.8 (2)	H10A—C10—H10C	109.5
O11—Mo3—O19	76.2 (2)	H10B—C10—H10C	109.5
O10—Mo3—O19	76.2 (2)	S6—C11—H11A	109.5
O17—Mo5—O14	104.5 (5)	S6—C11—H11B	109.5
O17—Mo5—O16	102.5 (4)	H11A—C11—H11B	109.5
O14—Mo5—O16	88.1 (3)	S6—C11—H11C	109.5
O17—Mo5—O4	104.5 (4)	H11A—C11—H11C	109.5
O14—Mo5—O4	87.4 (3)	H11B—C11—H11C	109.5
O16—Mo5—O4	152.9 (3)	S6—C12—H12A	109.5
O17—Mo5—O8	102.9 (5)	S6—C12—H12B	109.5
O14—Mo5—O8	152.6 (3)	H12A—C12—H12B	109.5
O16—Mo5—O8	85.9 (3)	S6—C12—H12C	109.5
O4—Mo5—O8	85.9 (3)	H12A—C12—H12C	109.5
O17—Mo5—O19	178.4 (4)	H12B—C12—H12C	109.5
O14—Mo5—O19	76.9 (2)	S7—C13—H13A	109.5
O16—Mo5—O19	76.6 (2)	S7—C13—H13B	109.5
O4—Mo5—O19	76.4 (2)	H13A—C13—H13B	109.5
O8—Mo5—O19	75.8 (2)	S7—C13—H13C	109.5
O28—Mo9—O23	104.6 (5)	H13A—C13—H13C	109.5
O28—Mo9—O26	103.5 (4)	H13B—C13—H13C	109.5
O23—Mo9—O26	88.0 (3)	S7—C14—H14A	109.5
O28—Mo9—O25^i^	102.9 (4)	S7—C14—H14B	109.5
O23—Mo9—O25^i^	86.8 (3)	H14A—C14—H14B	109.5
O26—Mo9—O25^i^	153.5 (3)	S7—C14—H14C	109.5
O28—Mo9—O22^i^	102.3 (5)	H14A—C14—H14C	109.5
O23—Mo9—O22^i^	153.1 (3)	H14B—C14—H14C	109.5
O26—Mo9—O22^i^	86.6 (3)	S8—C15—H15A	109.5
O25^i^—Mo9—O22^i^	86.4 (3)	S8—C15—H15B	109.5
O28—Mo9—O29	178.6 (4)	H15A—C15—H15B	109.5
O23—Mo9—O29	76.7 (2)	S8—C15—H15C	109.5
O26—Mo9—O29	76.8 (2)	H15A—C15—H15C	109.5
O25^i^—Mo9—O29	76.73 (19)	H15B—C15—H15C	109.5
O22^i^—Mo9—O29	76.4 (2)	S8—C16—H16A	109.5
O32—S1—C2	104.2 (5)	S8—C16—H16B	109.5
O32—S1—C1	105.1 (7)	H16A—C16—H16B	109.5
C2—S1—C1	97.7 (11)	S8—C16—H16C	109.5
O33—S2A—C4A	101.0 (15)	H16A—C16—H16C	109.5
O33—S2A—C3A	100.7 (7)	H16B—C16—H16C	109.5
C4A—S2A—C3A	97.5 (15)		

Symmetry codes: (i) −*x*, −*y*+1, −*z*; (ii) −*x*+1, −*y*+1, −*z*+1; (iii) *x*, *y*+1, *z*.

### Hydrogen-bond geometry (Å, º)

**Table:**

*D*—H···*A*	*D*—H	H···*A*	*D*···*A*	*D*—H···*A*
C2—H2*B*···O13^ii^	0.96	2.32	3.030 (14)	130
C6—H6*B*···O2^iv^	0.96	2.41	3.291 (18)	153
C11—H11*A*···O10	0.96	2.50	3.437 (15)	166
C14—H14*A*···O5^v^	0.96	2.46	3.386 (19)	163
C14—H14*B*···O20^vi^	0.96	2.36	3.255 (17)	154
C16—H16*C*···O14^v^	0.96	2.53	3.420 (17)	154

Symmetry codes: (ii) −*x*+1, −*y*+1, −*z*+1; (iv) *x*−1, *y*−1, *z*; (v) *x*, *y*−1, *z*; (vi) −*x*+1, −*y*+1, −*z*.
